# Evaluation of Food Homogenates on Cell Survival In Vitro

**DOI:** 10.1007/s12560-024-09586-3

**Published:** 2024-03-18

**Authors:** Dima Semaan, Liam O’Connor, Linda Scobie

**Affiliations:** Department of Biological and Biomedical Sciences, School of Health and Life Sciences, Glasgow Caledonian University, Glasgow, UK

**Keywords:** Food homogenates, Hepatitis E virus, A549, PLC/PRF/5, HepG2/C3A

## Abstract

A critical review on the approaches to assess the infectivity of the Hepatitis E virus (HEV) in food recommended that a cell culture-based method should be developed. Due to the observations that viral loads in food may be low, it is important to maximise the potential for detection of HEV in a food source in order to fully assess infectivity. To do so, would require minimal processing of any target material. In order to proceed with the development of an infectivity culture method that is simple, robust and reproducible, there are a number of points to address; one being to assess if food homogenates are cytotoxic to HEV susceptible target cells. Food matrices previously shown to have detectable HEV nucleic acid were selected for analysis and assessed for their effect on the percentage survival of three cell lines commonly used for infectivity assays. Target cells used were A549, PLC/PRF/5 and HepG2 cells. The results showed that, as expected, various food homogenates have differing effects on cells in vitro. In this study, the most robust cell line over a time period was the A549 cell line in comparison to HepG2, with PLC/PRF/5 cells being the most sensitive. Overall, this data would suggest that FH can be left in contact with A549 cells for a period of up to 72 h to maximise the potential for testing infection. Using food homogenates directly would negate any concerns over losing virus as a result of any additional processing steps.

**Supplementary Information:**

The online version contains supplementary material available at 10.1007/s12560-024-09586-3.

## Introduction

Hepatitis E (HEV) is a member of the *Hepeviridae* family encompassing eight different genotypes with many capable of infecting humans. HEV infection can lead to a wide range of clinical presentations from acute self-limiting infection to chronic infections, fulminant hepatitis and extrahepatic manifestations depending on the immune status, genotype and risk factors. The majority are asymptomatic and the majority of individuals with HEV infection recover completely. During hepatitis E outbreaks, the overall case-fatality rate is rare. (Treagus et al., 2021; Velavan & Pallerla, 2021; Primadharsini et al., 2021).

More than 20 million HEV infections occur annually in the world, with at least 3.3 million cases of acute illness and estimates of between 40 and 70 thousand deaths (World Health Organisation, 2019). In Europe, the predominant genotype is 3 and epidemiological evidence suggests that hepatitis E genotype 3 and 4 cases may be associated with the consumption of undercooked pork meat, offal and processed products such as sausages (Adlhoch et al., 2016; Treagus et al., 2021). Indeed, a UK study found HEV contamination in the UK pork production chain and that 10% of a small sample of retail pork sausages were contaminated with the virus (Berto et al., 2012). Furthermore, studies have confirmed the presence of HEV in shellfish in the UK (Crossan et al., 2014; O’Hara et al., 2018).

There are current uncertainties in many cases as to what this data represents and these studies have raised further questions such as, the prevalence of infectious HEV in foodstuffs, the effects of processing of these foods by industry and the effects of cooking and/or preparation by catering and consumers regarding the risk of infectivity of the virus.

Currently, there is no consistent or robust, validated test for HEV infectivity to assess this and a critical review on the approaches to assess the infectivity of the HEV virus (Cook et al., 2017) recommended that a cell culture-based method should be developed for use with food. It also identified other issues that may have affected the ability to develop a consistent method as differences in the length of time the virally contaminated sample is exposed to the cells and the concentration of the virus present. In most cases, the sample is only exposed to the cells for around 1 h and it has been shown that if the concentration is less that 1 × 10^3^ copies then infection is not established (Cook et al., 2017; Johne et al., 2014; Schemmerer et al., 2016; Treagus et al., 2021). This could be due to the complex replication cycle of the virus and the several quasi-enveloped states in which the virus can exist (Wißing et al., 2021). More recently, a number of articles have reported the development of culture systems with high viral loads to investigate the viral life cycle, inactivation and use of anti-virals (Schemmerer et al., 2019; Todt et al., 2020; Behrendt et al., 2022; Wißmann et al., 2023).

In order to proceed with the development of an infectivity culture method, there is also a requirement to assess if food matrices to be tested are detrimental to cell survival. A number of cell lines have been identified to be susceptible to HEV infection, albeit not formally validated (Schemmerer et al., 2016; Treagus et al., 2021). Many different cell lines have already been tested for their capability of supporting the replication of HEV in vitro. The most commonly utilised have been A549, PLC/PRF/5 and HepG2 (Schemmerer et al., 2019). Indeed, A549 and PLC/PRF/5 have been shown to be suitable for the isolation of swine and wild boar strains (Takahashi et al., 2012). As such, the aim of this pilot study is to produce data for relevant food products on their toxicity on these frequently used cell lines for HEV infectivity assays. In addition, we wished to see if cells would be affected by the time the food homogenate was in place and the best dilution of the FH for future analysis. The findings will allow us to address initial knowledge gaps in the development of an in vitro infectivity assay for food homogenates identified as positive for HEV nucleic acid.

## Methods

### Preparation of Food Homogenate

Selected food matrices were prepared from: strawberries (st), raspberries (Ras), lettuce (L), pork sausages 40% meat (PS1), pork sausages 97% meat (PS2), British pork chops (PC), smooth Brussels pâté (SBP), cooked mussels (CM), raw mussels (RM) and raw oysters (RO). All foods were purchased over the counter from supermarkets or outlets. Weights and volumes tested were scaled down to accommodate the large number of experiments required. Composition of the FH can be seen in Online Resource 1.

In brief, 30 mg (± 3 mg) sections composed of the entire food stuff without any prior treatment were placed in a tube containing sterile 1.0 mm dia. glass beads (BioSpec, Cambridge, UK) with 600 µl of sterile phosphate buffered saline (PBS pH7.4, Merck, Dorset, UK). Samples were homogenised at 50 Hz for 2.5 min in a TissueLyser LT (Qiagen, Surrey, UK). After homogenisation, samples were centrifuged at 10,000xg for 20 min at 4 °C. 500 µl of supernatant was transferred to a fresh tube after a 0.45uM (Sarstedt, Leicester, UK) filtration step and stored in aliquots at − 20 °C.

### Maintenance of Cells in Culture and Assay for Toxicity

To determine if the homogenates contained inhibitors or other content that is detrimental to the cells, the homogenates were added to selected cell lines that have been utilized previously to test infectivity of HEV in vitro (Schemmerer et al., 2016). Cells were purchased from ATCC (American Type Culture Collection): A549 (ATCC cell line number CCL-185), HepG2/C3A (ATCC cell line number HB-8065) and PLC/PRF/5 (ATCC cell line number CRL-8024). Cells were grown in 15 ml Gibco Dulbecco's modified Eagle's medium: Nutrient Mixture F-12, DMEM: F12 medium (ThermoFisher Scientific, Glasgow, UK) supplemented with 10% Gibco heat-inactivated Foetal bovine serum (FBS) (ThermoFisher Scientific) and 1% penicillin/Streptomycin 100X (Gibco, Fisher Scientific, 5000 U/ml) and 1% L-Glutamine (ThermoFisher Scientific) in T75 flasks (Corning, Dorset, UK) and maintained in culture at a density between 1–3 × 10^5^ cells/ml and sub-cultured every 5 days, until 70–80% confluent. Sub-culturing of cells was performed by trypsinisation with 2 ml of trypsin–EDTA 10X (0.5%, no phenol red (Gibco) at 37 °C. 8 ml of DMEM:F12 was added to the cells to neutralise the trypsin activity. The cells were centrifuged at 200×*g* for 5 min. Cell imaging was taken using an EVOS microscope (Invitrogen, ThermoFisher scientific). All cell lines and food homogenates were checked to be negative for HEV by PCR as described previously (Cook et al., 2022; Jothikumar et al., 2006).

To assess toxicity of the FH, a MTT assay (Invitrogen™ CyQUANT™ MTT Cell Viability Assay) was used to assess cell survival in vitro. In brief, A549, HepG2/C3A and PLC/PRF/5 cells were seeded in 96 well plates at a density of 1 × 10^4^ cells/well and cells were incubated at 37 °C with 5% CO_2_ for 24 h. Media was removed from the cells and the FH were added to the wells as undiluted (diluent was PBS (stock concentration of the FH is 50 mg/ml), a 1:2 dilution using cell culture media and a 1:5 dilution also with cell culture media then incubated at 37 °C with 5% CO_2_ for 24 h, 48 h and 72 h in duplicate.

At each duplicate time point, the supernatant was removed and 10 µl of MTT solution and 80 µl of cell culture medium were added to each well. Plates were then incubated at 37 °C for 3 h. After incubation, 100 µl of DMSO was added to each well. Plates were further incubated at room temperature in the dark for 10 min. Absorbance was read at OD = 590 nm using Epoque plate reader (BioTek, Cheshire, UK). PBS, dilutions with PBS and media and media only were used as controls for the effect on the cells.

### Statistical Analysis

Results are expressed as Mean ± SEM. statically significant differences were determined using PRISM version 4 Software by a Two-Way ANOVA Bonferroni Post-test with *p* < 0.001 as significant.

## Results

**Fig. 1:**
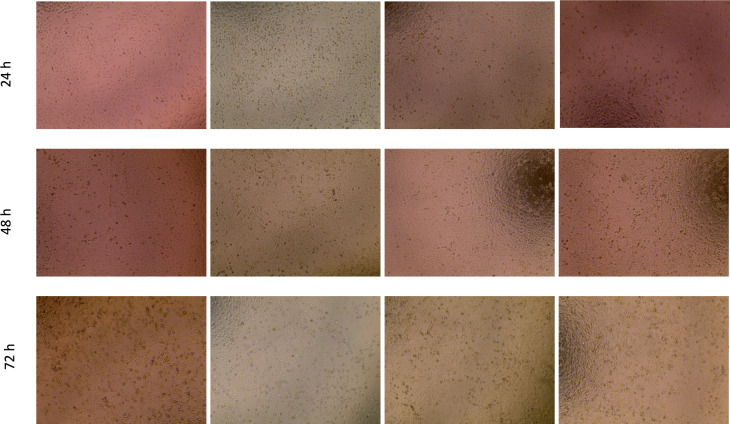
Viability and morphology of A549 cells after 24, 48 and 72 h following addition of undiluted, 1:2 dilution and 1:5 dilution of FH to the cells. Cells were seeded for 24 h in 96 well plate at 1 × 10^4^ cells/well in DMEM:F12 supplemented with 10% FBS at 37 °C with 5% CO_2_ (×10)

The 10 food homogenates (FH) were tested on A549, HepG2/C3A and PLC/PRF/5 cells as undiluted, 1:2 and 1:5 dilutions and the toxicity of these homogenates measured as cell death after 24 h, 48 h and 72 h. Morphological changes were noted in the cells by microscopy in addition to the MTT analysis. Figure 1 shows the effect of the control diluents on the morphology of the cell line A549. Images for PLC/PRF/5 and HepG2 provided in online resource 2 and 3 respectively.

**Fig. 2:**
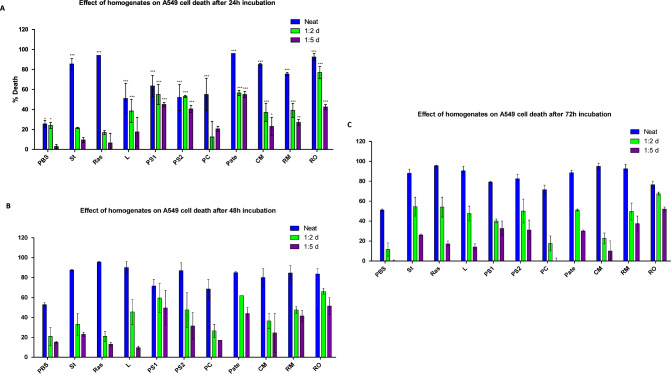
Cytotoxic effect of the food homogenates on A549 cells viability by MTT assay test. After 24 h of cell seeding in a 96-well plate, homogenates were added to the cells and incubated at 37 °C for 24 h (**A**), 48 h (**B**) and 72 h (**C**). Values are presented as percentage of cell death (± SEM) of n = 2. The data were analysed by two-Way ANOVA test, *p < 0.05, **p < 0.01 and ***p < 0.001 versus relevant control

Figure 2 illustrates the cell death at 24 (Fig. 2A), 48 (Fig. 2B) and 72 h (Fig. 2C) post treatment of A549 cells. For the dilution control, PBS, at 24 h, only 26%, 24% and 3% cell death was observed for undiluted, 1:2 and 1:5 dilutions respectively. As expected, at 48 and 72 h, percentage cell death increased in undiluted FH to an average of 52% (Fig. 2B, C) due to lack of nutrients on the cells. Both dilutions, were relatively unaffected over the time period. In contrast to this, all FH showed significant (*p* < 0.001) cell death when using undiluted FH at all time points.

A more complex picture was observed using the dilutions of the FH; no significant cell death (*p* > 0.05) was observed in the strawberry (St), raspberry (Ras), pork chops (PC) and lettuce (L) FH at a 1:5 dilution at either 24, 48 and 72 h. Cooked mussels also showed a non-significant level of cell death at a 1:5 dilution but only at 72 h (Fig. 2C).

**Fig. 3:**
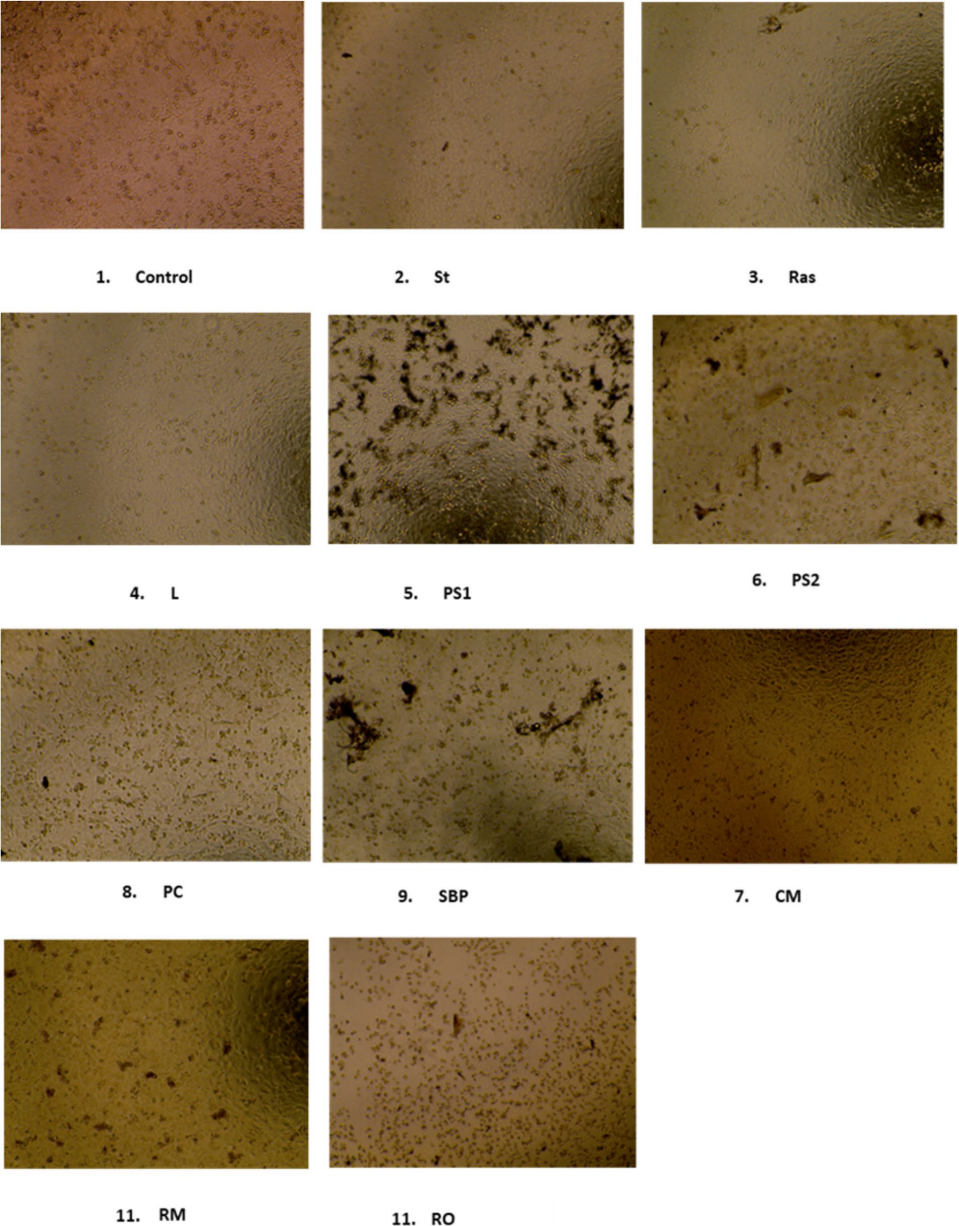
Morphology of A549 cells after 72 h following addition of 1:2 dilution of FH to the cells. Panel 1 is untreated cells, panels 2–11 are the FH. Cells were seeded for 24 h in 96 well plate at 1 × 10^4^ cells/well in DMEM:F12 supplemented with 10% FBS at 37 °C with 5% CO_2_ (×10)

In contrast the other FH did exhibit significant changes in the percentage cell death supported by the observed cell morphology in treated and untreated A549 cells at each time point and dilution. Cells treated with FH PS1, PS2, SBP, RM and RO show a cytopathic effect (CPE) on the A549 cells by exhibiting rounding up and clumping of the cells compared to the control (Fig. 3).

The same experiment was repeated for PLC/PRF/5 and HepG2 cells. For the PLC/PRF/5 cells at 24 h post treatment, significant cell death was seen only at a 1:5 dilution for pork sausage (PS1) and raw oyster (RO). Significant levels of cell death (*p* < 0.001) were observed at 48 and 72 h for all FH in PLC/PRF/5 cells by MTT assay (Online Resource 4A and 4B) regardless of dilution indicating these cells are more sensitive to toxic effects than A549 cells (Table 1). For the PLC/PRF/5 cells, CPE was observed for all of the FH at 72 h (Online Resource 4C) again supporting intolerance toxic effect of the FH. Likewise, a similar picture was observed for HepG2 cells, but only for the undiluted FH (*p* < 0.05); with dilution, significant cell death was only observed for FH SBP, RO, PS1 and St at 24 h (Online Resource 5A). At 48 and 72 h post treatment the cell death response to the FH became more variable suggesting that only certain FH were tolerated by HepG2 cells (Online Resource 5B and 5C).

**Table 1:** Summary of all average % cell death data collected from MTT assay on A549 cells over 3 days using undiluted FH, 1:2 and 1:5

			PBS	St	Ras	L	PS1	PS2	PC	SBP	CM	RM	RO
24 h	Undiluted FH	Mean	26	86	94	51	64	52	55	96	85	76	93
		± SEM	2	4	0	11	7	9	11	0	1	1	2
	1:2 dilution	Mean	24	22	17	39	55	53	13	57	37	39	77
		± SEM	2	0	1	8	7	1	11	2	6	5	4
	1:5 dilution	Mean	3	10	7	18	45	41	21	55	23	27	43
		± SEM	1	2	7	10	1	2	2	2	6	2	2
48 h	Undiluted FH	Mean	53	88	96	90	72	87	69	85	80	85	84
		± SEM	1	0	0	4	5	6	7	1	6	5	4
	1:2 dilution	Mean	21	33	21	46	60	48	27	62	37	48	66
		± SEM	6	8	4	9	10	12	5	0	5	2	2
	1:5 dilution	Mean	15	23	13	10	50	32	17	44	25	42	52
		± SEM	1	1	1	1	12	10	0	4	14	4	6
72 h	Undiluted FH	Mean	51	88	96	91	79	83	72	89	95	93	77
		± SEM	1	3	0	3	1	3	3	2	2	3	2
	1:2 dilution	Mean	12	55	54	48	40	50	18	51	23	50	68
		± SEM	5	7	7	5	1	8	5	1	4	6	1
	1:5 dilution	Mean	0	26	17	14	33	31	-4	30	10	38	52
		± SEM	1	1	2	2	5	7	5	1	7	5	1

Values are presented as mean results of 2 separate experiments done in duplicate. Values more than 20% death represent highly significant cell death indicated in Fig. 2 (***p < 0.001). Data for other cell lines can be found in Online Resource 6

## Summary and Discussion

Cultured cells can be deleteriously affected by substances co-extracted from pork products during sample treatment (Cook et al., 2017), and only three studies have reported the successful replication of HEV from pork products via cell culture (Takahashi et al., 2012; Berto et al., 2013a, b). Two of these studies utilised pig livers sold at retail; the other used a liver sausage sample for the inoculation (Berto et al., 2013a), however, one was a complex 3-D assay which may not be applicable in most laboratories.

As a number of foods have been shown to contain HEV nucleic acid, it would make sense to investigate any effects of these foods beyond pork products. It is apparent from this study, that varying FH has differing effects on cells in vitro. In terms of moving forward towards a suitable infectivity assay, it would seem reasonable to select A549 as the target cells to do this. A549 cells have been used previously in independent studies but have not been validated as repeatable and reproducible by an interlaboratory trial. CPE has been used as a parameter of HEV replication in these cells in numerous articles; these have used patient blood or faeces as a source of HEV which lack the components we would find in food (Cook et al., 2017; Johne et al., 2014; Schemmerer et al., 2019). Therefore, it is important to understand if the CPE is being produced by the FH or the virus itself. This would suggest that for FH, a direct measurement of the presence of replicating virus is a more substantial indication of infectivity. Indeed, adaptation of most of the published cell culture assays is required for use with food samples. (Cook et al., 2017).

The cytotoxic effect of the food homogenates (FH) was tested via the viability of the three cell lines over time. Cell death of more than 20% (cell viability less than 80% compared to the control untreated cells) was considered highly significant (*p* < 0.001). Overall, A549 was the most robust cell line in terms of tolerating the effects of FH over a time period of up to 72 h at a 1:2 dilution. PLC/PRF/5 were the most sensitive and subject to significant levels of cell death (> 20%) and the HepG2 appeared to be tolerant of only certain FH. A549 are human alveolar basal epithelial cells which is contrast to the liver derivation of the other two investigated. The reason for this variation in sensitivity to potential cytotoxic effects is unclear and could be multifactorial. All cell lines are immortal and are subject to differing culture conditions and growth patterns and may be affected by autophagy, however, this was not the aim of the study.

One other point that may contribute to the lack of consistency of methods is the contact exposure time of cells with the virus. Minimum contact time seen previously is an hour (Takahashi et al., 2012; Berto et al., 2013a, b). This begs the question as to whether the virus requires a longer incubation period, especially when present at lower concentrations such as those seen in food. Recent studies of HEV infectivity and replication in culture using human faecal sources already use a longer incubation time > 6 h in comparison to the food studies (Marion et al., 2020; Sayed et al., 2020).

There are also a number of reasons that could be related to loss of cells with specific food types. Soft fruits and leafy vegetables can be of a more acidic nature and it has been shown that these food groups can be contaminated with heavy metals such as cadmium and lead (Rusin et al., 2021). Pork sausage raw meat, pâté and cooked mussels contain high levels of fat (Online Resource 1), which also could contribute to cell death (Ebadi & Mazurak, 2014). It is unclear what effect any of this may have on the replication of HEV in the presence of FH and this remains to be investigated. If it can be demonstrated that there is no effect, then this will save additional processing steps in the assay and reduce loss of virus.

Specifically, the limits of detection of infectious HEV still need to be established for cell culture systems. Johne et al. (2016) demonstrated the titration of HEV infectivity in the A549/D3 cell line by immunofluorescence indicating a sensitive system for detection over a 4 log dilution. The use of differing HEV virus strains and virus titrations over a maximum of 4 log dilutions in the presence of relevant FH is required. Thus, the next steps to fulfilling the requirement for an infectivity assay will be to perform further experiments evaluating HEV replication with each specific FH utilising measurements of incubation/contact time with the cells, dilutions of the virus inoculate to the perceived low levels present in food, and detection by established PCR methods or immunofluorescence staining of viral proteins.

### Supplementary Information

Below is the link to the electronic supplementary material.

Supplementary file1 (DOCX 5001 kb)
