# Supplementary material for: Evaluation of Food Homogenates on Cell Survival In Vitro
Source: Food Environ Virol. 2024 Mar 18;16(2):253–60. doi: 10.1007/s12560-024-09586-3 (PMC11186945; doi:10.1007/s12560-024-09586-3)
Supplement: Supplementary file 1 — Supplementary file1 (DOCX 5001 kb) [file 12560_2024_9586_MOESM1_ESM.docx]

**Online Resource 1.** Summary table of the food homogenates composition.

|  |  | |  | |  | |  | |  | |  | |  | |
| --- | --- | --- | --- | --- | --- | --- | --- | --- | --- | --- | --- | --- | --- | --- |
|  | **per** | **Energy** | | **Fat** | | **Saturate** | | **Carbs** | | **Sugars** | **Fibre** | **Protein** | | **Salt** |
| Strawberry | *100g* | 128kJ / 30kcal | | 0.1g | | <0.1g | | 6.0g | | 6.0g | 1.1g | 0.8g | | 0.1g |
| Raspberry | *100g* | 133kJ / 32kcal | | 0.3g | | 0.1g | | 4.6g | | 4.6g | 2.5g | 1.4g | | 0g |
| Lettuce | *100g* | 48kJ / 11kcal | | 0.1g | | <0.1g | | 1.4g | | 1.4g | 1.5g | 1.2g | | 0.1g |
| Pork sausages (40% meat) | *100g* | 460kJ/240kcal | | 15g | | 5.5g | | 14g | | 1.2g | - | 12g | | 2.3g |
| Pork sausages (97% meat) | *100g* | 1182kJ/288kcal | | 22g | | 7.6g | | 1.2g | | 0.1g | - | 20g | | 1.9g |
| Pork chops | *100g* | 1119kJ / 270kcal | | 21.7g | | 8.0g | | 0g | | 0g | 0g | 18.6g | | 0.3g |
| Brussels Paté | *100g* | 372kJ/90kcal | | 7.1g | | 2.7g | | 0g | | 1.1g | 0g | - | | 0.57g |
| Cooked Mussels | *100g* | 401kJ / 95kcal | | 2.6g | | 0.5g | | 4.3g | | 0g | 0g | 13.7g | | 0.8g |
| Raw mussels | *100g* | 86kcal | | 2.26g | | 0.4g | | 3.6g | | 0g | 0g | 12g | | - |
| Raw oysters | *100g* | 72kcal | | 1.5g | | 0g | | 3g | | 0g | 0g | 7g | | 0g |
|  |  |  | |  | |  | |  | |  |  |  | |  |

**Online Resource 2.** Viability and morphology of PLC/PRF/5 cells after 24, 48 and 72h following addition of undiluted, 1:2 dilution and 1:5 dilution of FH to the cells. Cells were seeded for 24h in 96 well plate at 1x10^4^cells/well in DMEM:F12 supplemented with 10% FBS at 37^°^C in 5% CO_2_ (x10).

ontrol untreated PLC/PRF/5 PLC/PRF/5 undiluted FH PLC/PRF/5 1:2 dilution PLC/PRF/5 1:5 dilution


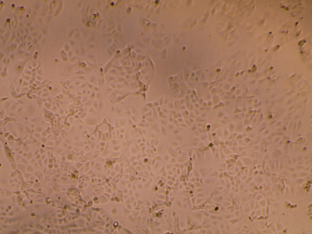

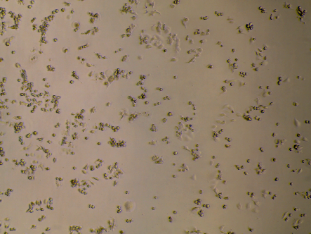

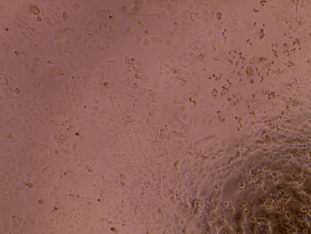

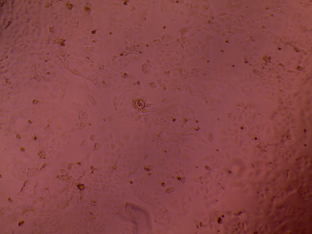


24 h


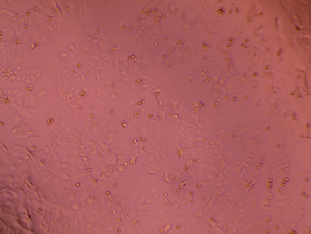

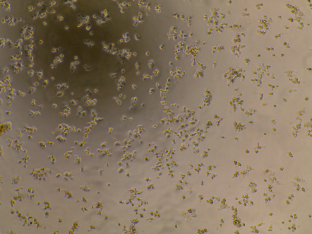

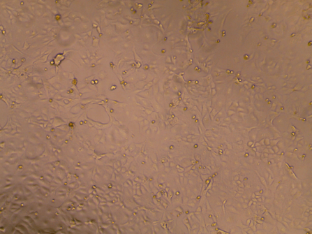

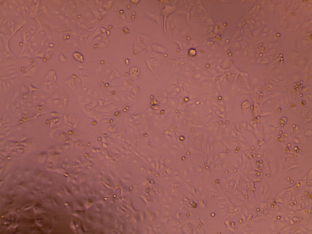


48 h


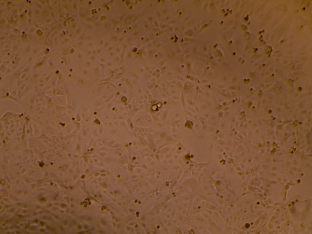

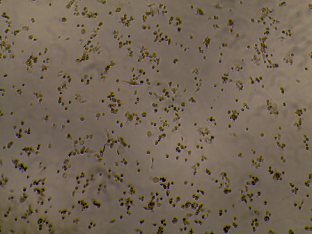

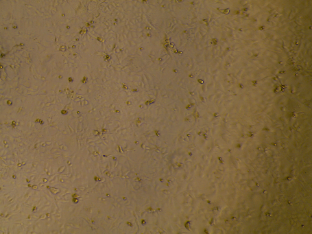

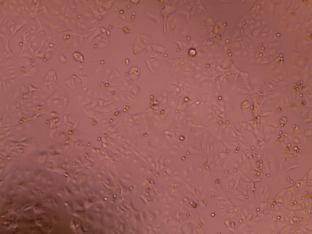


72h

**Online Resource 3.** Viability and morphology of HepG2 cells after 24, 48 and 72h following addition of undiluted, 1:2 dilution and 1:5 dilution of FH to the cells. Cells were seeded for 24h in 96 well plate at 1x10^4^cells/well in DMEM:F12 supplemented with 10% FBS at 37^°^C with 5% CO_2_ (x10).

**Online Resource 4.** Cytotoxic effect of the food homogenates on PLC/PRF/5 cells viability by MTT assay test. After 24h of cell seeding in a 96-well plate, homogenates were added to the cells and incubated at 37^°^C for 24h (A), 48h (B) and 72h (C). Then MTT assay was followed. Values are presented as percentage of death (±SEM) of n=2. The data were analysed by two-Way ANOVA test, **P*<0.05, ***P*<0.01 and ****P*<0.001 versus relevant control.

**A**

**B**

**C**

**Online Resource 5.**  Cytotoxic effect of the food homogenates on HepG2 cells viability by MTT assay test. After 24h of cell seeding in a 96-well plate, homogenates were added to the cells and incubated at 37^°^C for 24h (A), 48h (B) and 72h (C). Then MTT assay was followed. Values are presented as percentage of death (±SEM) of n=2. The data were analysed by two-Way ANOVA test, **P*<0.05, ***P*<0.01 and ****P*<0.001 versus relevant control.

**A**

**B**

**C**

**Online Resource 6.** Summary Table of all data collected from MTT assay of the percentage cell death of the three cell lines over 3 days using undiluted, 1:2 and 1:5 dilution of the FH. Values are presented as mean results of 2 experiments in duplicates of the three cell lines. A. Effect of FH on A549 cell death, B. Effect of FH on PLC/PRF/5 cell death and C. Effect of FH on HepG2 cell death. Values more than 20% death represent the highly significant cell death.

| A. | A549 |  | | | | | | | | | | | |
| --- | --- | --- | --- | --- | --- | --- | --- | --- | --- | --- | --- | --- | --- |
|  | | | **PBS** | **St** | **Ras** | **L** | **PS1** | **PS2** | **PC** | **SBP** | **CM** | **RM** | **RO** |
| 24h | **Undiluted FH** | **Mean** | 26 | 86 | 94 | 51 | 64 | 52 | 55 | 96 | 85 | 76 | 93 |
|  |  | **±SEM** | 2 | 4 | 0 | 11 | 7 | 9 | 11 | 0 | 1 | 1 | 2 |
|  | **1:2 dilution** | **Mean** | 24 | 22 | 17 | 39 | 55 | 53 | 13 | 57 | 37 | 39 | 77 |
|  |  | **±SEM** | 2 | 0 | 1 | 8 | 7 | 1 | 11 | 2 | 6 | 5 | 4 |
|  | **1:5 dilution** | **Mean** | 3 | 10 | 7 | 18 | 45 | 41 | 21 | 55 | 23 | 27 | 43 |
|  |  | **±SEM** | 1 | 2 | 7 | 10 | 1 | 2 | 2 | 2 | 6 | 2 | 2 |
| 48h | **Undiluted FH** | **Mean** | 53 | 88 | 96 | 90 | 72 | 87 | 69 | 85 | 80 | 85 | 84 |
|  |  | **±SEM** | 1 | 0 | 0 | 4 | 5 | 6 | 7 | 1 | 6 | 5 | 4 |
|  | **1:2 dilution** | **Mean** | 21 | 33 | 21 | 46 | 60 | 48 | 27 | 62 | 37 | 48 | 66 |
|  |  | **±SEM** | 6 | 8 | 4 | 9 | 10 | 12 | 5 | 0 | 5 | 2 | 2 |
|  | **1:5 dilution** | **Mean** | 15 | 23 | 13 | 10 | 50 | 32 | 17 | 44 | 25 | 42 | 52 |
|  |  | **±SEM** | 1 | 1 | 1 | 1 | 12 | 10 | 0 | 4 | 14 | 4 | 6 |
| 72h | **Undiluted FH** | **Mean** | 51 | 88 | 96 | 91 | 79 | 83 | 72 | 89 | 95 | 93 | 77 |
|  |  | **±SEM** | 1 | 3 | 0 | 3 | 1 | 3 | 3 | 2 | 2 | 3 | 2 |
|  | **1:2 dilution** | **Mean** | 12 | 55 | 54 | 48 | 40 | 50 | 18 | 51 | 23 | 50 | 68 |
|  |  | **±SEM** | 5 | 7 | 7 | 5 | 1 | 8 | 5 | 1 | 4 | 6 | 1 |
|  | **1:5 dilution** | **Mean** | 0 | 26 | 17 | 14 | 33 | 31 | -4 | 30 | 10 | 38 | 52 |
|  |  | **±SEM** | 1 | 1 | 2 | 2 | 5 | 7 | 5 | 1 | 7 | 5 | 1 |

| B. | PLC/PRF/5 |  | | | | | | | | | | | |
| --- | --- | --- | --- | --- | --- | --- | --- | --- | --- | --- | --- | --- | --- |
|  | | | **PBS** | **St** | **Ras** | **L** | **PS1** | **PS2** | **PC** | **SBP** | **CM** | **RM** | **RO** |
| 24h | **Undiluted FH** | **Mean** | 18 | 73 | 91 | 94 | 75 | 65 | 17 | 78 | 67 | 71 | 89 |
|  |  | **±SEM** | 6 | 7 | 2 | 2 | 1 | 10 | 2 | 8 | 11 | 1 | 1 |
|  | **1:2 dilution** | **Mean** | 14 | 21 | 49 | 38 | 64 | 53 | 2 | 67 | 31 | 25 | 61 |
|  |  | **±SEM** | 4 | 5 | 11 | 5 | 1 | 5 | 5 | 7 | 5 | 2 | 6 |
|  | **1:5 dilution** | **Mean** | -4 | 12 | 12 | 17 | 32 | 16 | -1 | 46 | 3 | 7 | 26 |
|  |  | **±SEM** | 1 | 6 | 2 | 1 | 0 | 7 | 1 | 8 | 4 | 5 | 0 |
| 48h | **Undiluted FH** | **Mean** | 87 | 83 | 95 | 90 | 86 | 77 | 68 | 91 | 82 | 89 | 86 |
|  |  | **±SEM** | 4 | 4 | 1 | 4 | 3 | 8 | 11 | 2 | 7 | 3 | 5 |
|  | **1:2 dilution** | **Mean** | 22 | 49 | 74 | 45 | 58 | 49 | 41 | 85 | 75 | 60 | 75 |
|  |  | **±SEM** | 1 | 7 | 7 | 0 | 2 | 4 | 5 | 7 | 5 | 2 | 1 |
|  | **1:5 dilution** | **Mean** | 9 | 32 | 42 | 32 | 53 | 39 | 29 | 78 | 42 | 41 | 62 |
|  |  | **±SEM** | 6 | 1 | 1 | 3 | 3 | 1 | 2 | 6 | 5 | 5 | 1 |
| 72h | **Undiluted FH** | **Mean** | 74 | 85 | 95 | 96 | 94 | 92 | 95 | 96 | 95 | 92 | 94 |
|  |  | **±SEM** | 3 | 4 | 1 | 1 | 2 | 2 | 1 | 1 | 1 | 2 | 1 |
|  | **1:2 dilution** | **Mean** | 22 | 66 | 71 | 96 | 95 | 92 | 54 | 94 | 71 | 63 | 84 |
|  |  | **±SEM** | 6 | 7 | 10 | 2 | 2 | 3 | 5 | 2 | 4 | 2 | 6 |
|  | **1:5 dilution** | **Mean** | 4 | 58 | 57 | 91 | 95 | 62 | 38 | 86 | 58 | 49 | 58 |
|  |  | **±SEM** | 0 | 11 | 11 | 2 | 1 | 10 | 1 | 4 | 7 | 3 | 5 |

| C. | HepG2 |  | | | | | | | | | | | |
| --- | --- | --- | --- | --- | --- | --- | --- | --- | --- | --- | --- | --- | --- |
|  | | | **PBS** | **St** | **Ras** | **L** | **PS1** | **PS2** | **PC** | **SBP** | **CM** | **RM** | **RO** |
| 24h | **Undiluted FH** | **Mean** | 23 | 85 | 93 | 79 | 67 | 55 | 38 | 76 | 49 | 58 | 79 |
|  |  | **±SEM** | 3 | 0 | 1 | 12 | 7 | 6 | 7 | 4 | 4 | 10 | 8 |
|  | **1:2 dilution** | **Mean** | 20 | 30 | 31 | 30 | 46 | 9 | 2 | 52 | 7 | 5 | 54 |
|  |  | **±SEM** | 0 | 2 | 3 | 10 | 14 | 3 | 5 | 3 | 12 | 12 | 1 |
|  | **1:5 dilution** | **Mean** | 14 | 26 | 13 | 16 | 30 | 7 | 2 | 40 | -5 | 2 | 42 |
|  |  | **±SEM** | 1 | 1 | 4 | 4 | 4 | 2 | 3 | 1 | 5 | 2 | 9 |
| 48h | **Undiluted FH** | **Mean** | 60 | 92 | 95 | 82 | 84 | 74 | 64 | 88 | 93 | 94 | 97 |
|  |  | **±SEM** | 2 | 1 | 0 | 11 | 10 | 2 | 0 | 0 | 2 | 2 | 0 |
|  | **1:2 dilution** | **Mean** | 24 | 64 | 48 | 68 | 57 | 36 | 8 | 67 | 40 | 59 | 66 |
|  |  | **±SEM** | 3 | 3 | 7 | 14 | 3 | 5 | 5 | 0 | 1 | 3 | 7 |
|  | **1:5 dilution** | **Mean** | 3 | 24 | 12 | 21 | 27 | 8 | 1 | 45 | 28 | 35 | 46 |
|  |  | **±SEM** | 2 | 2 | 5 | 6 | 9 | 7 | 4 | 4 | 13 | 10 | 8 |
| 72h | **Undiluted FH** | **Mean** | 80 | 90 | 95 | 69 | 78 | 74 | 81 | 90 | 98 | 86 | 97 |
|  |  | **±SEM** | 0 | 1 | 1 | 2 | 3 | 1 | 5 | 2 | 0 | 1 | 0 |
|  | **1:2 dilution** | **Mean** | 32 | 74 | 50 | 49 | 58 | 47 | 15 | 61 | 44 | 67 | 73 |
|  |  | **±SEM** | 4 | 4 | 3 | 10 | 7 | 1 | 2 | 3 | 7 | 4 | 5 |
|  | **1:5 dilution** | **Mean** | 7 | 44 | 22 | 19 | 43 | 14 | 15 | 56 | 21 | 41 | 61 |
|  |  | **±SEM** | 5 | 2 | 8 | 11 | 4 | 7 | 8 | 6 | 2 | 12 | 6 |
